# Implementing psilocybin-assisted therapy in palliative care settings: A survey of stakeholders

**DOI:** 10.1177/02692163261446141

**Published:** 2026-05-19

**Authors:** Louis Plourde, Sue-Ling Chang, Olivia Nguyen, Nicolas Garel, Houman Farzin, Jean-François Stephan, Jean-Sébastien Fallu, Michel Dorval, P3A Research Group

**Affiliations:** 1 Faculty of Pharmacy, Université Laval, QC, Canada; 2 Oncology Division, CHU de Québec-Université Laval Research Centre, QC, Canada; 3 Réseau québécois de recherche en soins palliatifs et de fin de vie (RQSPAL), QC, Canada; 4 Faculty of Medicine, Université de Montréal, QC, Canada; 5 CIUSSS Nord-de-l’Île-de-Montréal, QC, Canada; 6 Société québécoise des médecins de soins palliatifs (SQMDSP), Montréal, QC, Canada; 7 Department of Psychiatry and Addictology, Faculty of Medicine, Université de Montréal, QC, Canada; 8 Centre hospitalier de l’Université de Montréal Research Centre (CRCHUM), QC, Canada; 9 Lady Davis Institute for Medical Research, Jewish General Hospital, Montréal, QC, Canada; 10 Faculty of Medicine and Health Sciences, McGill University, Montréal, QC, Canada; 11 Private Practice, Montréal, QC, Canada; 12 School of Psychoeducation, Université de Montréal, QC, Canada; 13 Center for Public Health Research (CReSP), Montréal, QC, Canada; 14 CISSS Chaudière-Appalaches Research Center, Lévis, QC, Canada

**Keywords:** psilocybin, hallucinogens, palliative care, surveys and questionnaires, attitude of health personnel

## Abstract

**Background:**

While the adoption of psilocybin-assisted therapy for existential distress offers promising support for patients with life-threatening illnesses, implementing this intervention into palliative care settings presents significant real-world challenges.

**Aim:**

To examine palliative care stakeholders’ knowledge and attitudes regarding psilocybin-assisted therapy, and identify barriers and facilitators to its implementation.

**Design:**

We conducted a cross-sectional online survey between April 15 and December 18, 2024. The survey assessed perceived knowledge, attitudes, and perceived barriers and facilitators to the effective integration of psilocybin-assisted therapy into palliative care settings.

**Setting/participants:**

One hundred and twenty-one adults involved in palliative care (physicians, other healthcare professionals, caregivers, and managers) were recruited from Canada’s four most populous provinces: Québec, Ontario, Alberta, and British Columbia.

**Results:**

Forty-three percent of stakeholders reported having good knowledge of psilocybin’s potential benefits and risks. Attitudes towards psilocybin-assisted therapy were predominantly non-favourable (61%), yet varied across occupational groups (*p* < 0.0001), with 95% of physicians reporting favourable attitudes. The lack of trained healthcare providers was viewed as the primary barrier to implementation. Key facilitators included further research and developing standardised intervention protocols. Sixty-eight percent of stakeholders endorsed the introduction of psilocybin-assisted therapy during the early stages of the illness trajectory.

**Conclusions:**

Translating the potential of psilocybin-assisted therapy for existential distress from clinical trials into palliative care settings requires careful consideration and collaboration with stakeholders. Given the significant divergence in perspectives between clinical and non-clinical groups, tailored interprofessional education could help build shared understanding and support effective implementation. Being conducted in Canada, transferability to different regulatory frameworks may be limited.

**What is already known on this topic?**

- Regulatory frameworks in select jurisdictions have begun to accommodate psilocybin-assisted therapy to address existential distress in patients with life-threatening illnesses.
- Prior research indicates that clinicians generally hold favourable views towards psilocybin-assisted therapy, though concerns persist regarding training, costs, institutional barriers, and the risk-benefit balance in vulnerable populations.

**What this study adds?**

- By engaging previously overlooked palliative care stakeholders (caregivers and managers), this study reveals contrasting perspectives that broaden the discourse beyond a clinician-centric view.
- Attitudes towards the safety and therapeutic potential of psilocybin-assisted therapy were less favourable than previously reported. Clinical background and direct patient care responsibilities (notably among physicians) appear to foster more positive perceptions of the intervention.

**How this study might affect research, practice, or policy**

- Highlights the need for interprofessional education initiatives to bridge knowledge gaps and align stakeholder attitudes.
- Informs strategic priorities, including the development of standardised intervention protocols, the scaling up of certified training programmes, and the establishment of dedicated delivery settings.
- Supports the early introduction of psilocybin-assisted therapy in the course of life-threatening illnesses.

## Background

Over the past decade, psilocybin-assisted therapy has gained recognition as a safe^1,2^ and promising^3–8^ intervention to treat depression, anxiety and existential distress^9–11^ in patients with life-threatening illnesses. Despite the low certainty of evidence regarding clinical outcomes^12^ and pervasive legal restrictions worldwide, this serotonergic psychedelic found in *Psilocybe* mushrooms is advancing from clinical trials into innovative healthcare settings. Australia, Canada, Switzerland, and select U.S. jurisdictions have spearheaded the adoption of psilocybin-assisted therapy into formal or regulated clinical pathways,^13–16^ while the Czech Republic, New Zealand, and Germany have more recently followed suit.^17–20^ While these regulatory shifts are encouraging, deploying this novel, complex intervention raises significant challenges,^21^ notably in palliative medicine.^22,23^ Similarly, the use of lysergic acid diethylamide (LSD) is gaining empirical traction as a potentially beneficial therapeutic option to treat anxiety and comorbid depression symptoms in patients with life-threatening illnesses.^24–26^

Existing literature provides a substantive overview of healthcare providers’ perspectives on the clinical potential and practical implications of psilocybin- and other psychedelic-assisted therapies, reflecting a prevailing sense of cautious optimism.^27–32^ Common concerns include the scarcity of trained professionals to deliver the intervention, its associated financial burden, and the potential risks posed by patient-specific contraindications.^28^ In the field of palliative medicine, more specifically, the broadly positive attitudes of professionals is tempered with a clear call for rigorous clinical trials to further explore how the intervention might complement standard medical protocols.^33^ Perceived misalignment of psilocybin-assisted therapy with established care practices has prompted key concerns about institutional and organisational constraints, as well as uncertainties in balancing risks with benefits in this particularly vulnerable population.^34–36^ Despite these valuable insights, significant gaps remain in understanding the real-world challenges of deploying psilocybin-assisted therapy in the context of palliative care to alleviate patients’ existential distress. By focussing almost exclusively on clinicians’ perspectives, prior research has overlooked other critical voices in the field: those of caregivers and managers, who are well-positioned to shed light on institutional and infrastructural considerations.

The Canadian context of psilocybin-assisted therapy in palliative care is particularly salient, as insights drawn from this setting may have implications for international practice and policy, with due consideration of cross-jurisdictional regulatory differences. Having rapidly evolved since the federal health agency adopted regulatory pathways allowing the clinical use of psilocybin and MDMA (3,4-methylenedioxymethamphetamine) for patients unresponsive to conventional treatments, it offers a clear lens on the various conditions that may undermine successful implementation. The country’s first personal exemptions were granted in 2020 to four cancer patients, allowing them to legally possess and use psilocybin to alleviate their existential distress.^14^ As of 2022, physicians and specialised nurse practitioners can request these otherwise restricted substances through the Special Access Program, in order to treat patients for whom available treatments have proven ineffective, are unsuitable, or are unavailable.^37,38^ Within the scope of 3 years, a total of 95 requests have been approved for end-of-life distress, representing an approval rate of 87%.^39^ Despite Canada’s groundbreaking regulatory amendment, the static and low demand for psilocybin-assisted therapy, as well as its critically limited accessibility, points to enduring challenges hindering the passage of this promising intervention into real-world scenarios.^39,40^

The present study examined the perspectives of Canadian palliative care stakeholders regarding the adoption and delivery of psilocybin-assisted therapy for existential distress. Drawing on constructs from implementation science,^41^ such as the updated Consolidated Framework for Implementation Research^42,43^ and the Implementation Outcomes Framework,^44^ this study was designed to assess acceptability and identify perceived barriers and key facilitators that could support the effective integration of this novel intervention into clinical settings. These findings are intended to inform future strategic planning and policy initiatives aimed at optimising accessibility and promoting equity for individuals facing life-threatening illnesses.

## Methods

### Study design

We conducted a cross-sectional online survey between April 15 and December 18, 2024. The results are presented in accordance with the Checklist for Reporting Results of Internet E-Surveys (CHERRIES)^45^ (see Supplemental material).

### Sampling and data collection

Data for this study were collected through two distinct recruitment strategies. The study included adults involved in palliative care, such as physicians, other healthcare professionals, caregivers and managers. Patients were not eligible for this study.

***Sample 1***: The initial recruitment targeted residents of the province of Québec. Convenience sampling was carried out through advertisements in newsletters and bulletins distributed through palliative care networks. Leaflets with a survey link were also distributed at palliative care related conferences and meetings. The questionnaire was administered online between April 15 and November 30, 2024, using the REDCap platform hosted by Université Laval.
***Sample 2***: To strengthen the study sample, additional recruitment was conducted in the four most populous Canadian provinces: Québec, Ontario, Alberta, and British Columbia. We reached out to participants from an earlier online survey^46^ who had (1) indicated prior exposure to palliative care, and (2) consented to be recontacted for future research on the topic. Recruitment and data collection, done between October 31 and December 18, 2024, were overseen by the research company Leger. A link to the survey questionnaire was emailed to potential participants.

The anonymous survey, available in English and French, was identical across both samples. Participation was voluntary, and all respondents provided informed consent. Only completed questionnaires were analysed.

### Measures

The instrument consisted of 34 closed-ended items and 1 open-ended question (see Supplemental material). To facilitate participants’ understanding, comprehensive definitions (e.g. palliative care, existential distress, psilocybin-assisted therapy) and contextual information (e.g. Canada’s Special Access Program) were provided throughout the questionnaire. A series of descriptive items collected detailed information about respondents’ personal and professional profiles, including age, gender, occupation, number of years involved in palliative care, primary context of involvement, weekly hours of patient contact, and previous use of psilocybin.

Individuals involved in palliative care were categorised into four subgroups: (1) Physicians, (2) Other professionals (including nurse clinicians, licenced practical nurses, specialised nurse practitioners, psychologists, pharmacists, physiotherapists, occupational therapists, spiritual care providers, social workers), (3) Caregivers (beneficiary attendants, caregivers, volunteers, patient partners), and (4) Managers (including administrators).

Stakeholders perceived knowledge regarding the potential benefits and risks of psilocybin, as well as their attitudes towards its safety and use in treating existential distress, were assessed using five-point Likert scale items (1 = “Strongly disagree” to 5 = “Strongly agree”). Two items pertained to perceived knowledge, and four to stakeholders’ attitudes. A five-option item evaluated the prospective acceptability^44^ of psilocybin-assisted therapy among stakeholders (“Do you believe psilocybin-assisted therapy is a reasonable medical choice for someone with a serious and incurable illness suffering from existential distress?”). Response options included three variations of “Yes,” one “No” option with open-text justification, and one “Uncertain” option. Additionally, an ordinal categorical item measured the perceived level of professional liability risk associated with psilocybin-assisted therapy delivery (“No risk,” “Low risk,” “Moderate risk,” “High risk”).

Challenges (barriers) to implementing psilocybin-assisted therapy into palliative care settings and actionable measures (facilitators) for its successful deployment were identified through sections containing nominal and multiple-response categorical items, measuring their perceived importance among stakeholders. The questionnaire underwent pretesting with 16 volunteers to ensure proper functionality and to establish an estimated completion time (10–15 min). Minor adjustments were made based on their feedback.

### Data analysis

Six items assessing participants perceived knowledge and attitudes towards psilocybin-assisted therapy underwent principal component analysis with Varimax rotation to examine their underlying structure and identify coherent item groupings. Two distinct factors emerged, reflecting perceived knowledge (two items; Cronbach’s α = 0.89) and attitudes (four items; Cronbach’s α = 0.93), confirming that the items clustered meaningfully within these dimensions. Composite scores (shown in Supplemental Table 2, Supplemental materials) were calculated by summing item responses for each factor, yielding ranges of 2–10 for perceived knowledge and 4–20 for attitudes. To enhance interpretability and ensure adequate cell sizes for subgroup comparisons, composite scores were dichotomised using a threshold of >60% of the maximum possible score, corresponding to >6/10 for knowledge (“Good” vs “Limited”) and >12/20 for attitudes (“Favourable” vs “Non-favourable”). This approach was employed to identify participants with distinctly positive perceptions, minimising neutral or borderline responses.

Statistically significant associations between survey outcomes (dichotomous and categorical items) and occupational groups (Physicians, Other professionals, Caregivers, Managers) were assessed with two-sided χ^2^ tests using a *p*-value threshold of ⩽0.05. Since our sample consisted of volunteers, our results are unweighted. To assess the robustness of the findings, sensitivity analyses were conducted using logistic regression models adjusting for age and gender for the primary outcomes (e.g. acceptability and attitudes towards psilocybin-assisted therapy). As these variables were not significantly associated with the outcomes and yielded results comparable to the unadjusted models, only unadjusted analyses are presented. Data management and statistical analyses were performed using SAS version 9.4 (SAS Institute).

## Results

### Sample characteristics

A total of 163 respondents completed the survey across the 2 recruitment strategies. In Sample 1, 48 individuals responded to the questionnaire; 5 (researchers, students) did not meet the inclusion criteria and were excluded, yielding a final sample of 43 participants. In Sample 2, an invitation was sent to 246 potential participants; 3 declined, 15 did not complete the questionnaire, and 115 completed it. Of these, 37 were excluded due to unclear involvement in palliative care, resulting in 78 participants who met the inclusion criteria. The final combined sample, therefore, comprised 121 participants. Overall sample characteristics are presented in Table 1, while descriptive statistics for each sample are provided in Supplemental Table 1.

**Table 1.** Sample characteristics.

	Physicians		Other professionals		Caregivers		Managers		Global	
Characteristic	*n*	%	*n*	%	*n*	%	*n*	%	*n*	%
Total	20	100	48	100	36	100	17	100	121	100
Age^b^										
18–34	3	15	18	38	3	8	2	12	26	21
35–54	9	45	23	48	10	28	11	65	53	44
⩾55	8	40	7	15	23	64	4	24	42	35
Gender^a^										
Woman	12	60	41	85	19	53	8	47	80	66
Man	7	35	7	15	16	44	9	53	39	32
Non-binary	0	0	0	0	1	3	0	0	1	1
Missing	1	5	-	-	-	-	-	-	1	1
Years involved in the field of palliative care^a^										
0–2	1	5	13	27	13	36	6	35	33	27
3–10	6	30	18	38	11	31	7	41	42	35
>10	13	65	17	35	6	17	4	24	40	33
Missing	-	-	-	-	6	17	-	-	6	5
Primarily involved in^a^										
Hospital	14	70	19	40	5	14	4	24	42	35
Outpatient clinic	1	5	5	10	1	3	0	0	7	6
Palliative care home	4	20	4	8	2	6	3	18	13	11
Patients’ homes	1	5	11	23	16	44	2	12	30	25
Long-term care centre	0	0	3	6	2	6	1	6	6	5
Academia	0	0	1	2	0	0	3	18	4	3
Missing	-	-	5	10	10	28	4	24	19	16
Approximate number of hours per week in contact with people in palliative care^b^										
0	0	0	4	8	11	31	4	24	19	16
1–10	2	10	19	40	14	39	2	12	37	31
11–20	5	25	9	19	4	11	6	35	24	20
>20	13	65	13	27	5	14	4	24	35	29
Missing	-	-	3	6	2	6	1	6	6	5
Lifetime psilocybin use										
Yes	4	20	12	25	6	17	7	41	29	24
No	15	75	36	75	30	83	9	53	90	74
Missing	1	5	-	-	-	-	1	6	2	2

a *p*-value ⩽0.05.

b *p*-value <0.0001.

Participants were distributed across four occupational groups: 20 physicians, 48 other professionals (including 35 nurses), 36 caregivers, and 17 managers. The global sample was balanced across age groups. Women accounted for 66% of participants, with the highest proportion among other professionals (85%; *p* = 0.0121). One participant (1%) identified as non-binary.

Among physicians (*n* = 20), 65% had more than 10 years’ experience in palliative care. Most worked in hospitals (70%) and maintained extensive patient contact, with 65% providing over 20 h per week of direct care. The experience of other professionals (*n =* 48) was more evenly distributed, with roughly one-third falling in each range of years of involvement. While hospitals were their most common workplace (40%), a notable proportion worked in patients’ homes (23%). Caregivers (*n =* 36) were, on average, older than other occupational groups (*p* < 0.0001), with 64% aged ⩾55 years. Their primary care setting was patients’ homes (44%). Managers (*n =* 17) were mostly aged 35–54 years (65%) and worked across diverse settings, with no single environment predominating.

Overall, 24% of participants reported having used psilocybin at least once in their lifetime (either for therapeutic or recreational purposes), with no significant difference between occupational groups.

### Perceived knowledge and attitudes

As shown in Table 2, 43% of stakeholders considered having good knowledge of the potential benefits and risks of psilocybin. Attitudes towards psilocybin-assisted therapy varied significantly across occupational groups (*p* < 0.0001), with physicians being markedly more favourable (95%) compared to other occupations (individual Likert item scores and composite mean scores provided in Supplemental Table 2). Most participants (85%) considered psilocybin-assisted therapy to be a reasonable medical choice for individuals experiencing existential distress due to a serious, incurable illness. Opinions were divided regarding the degree of professional liability risk posed by psilocybin-assisted therapy.

**Table 2.** Perceived knowledge and attitudes.

	Physicians		Other professionals		Caregivers		Managers		Global	
Item	*n*	%	*n*	%	*n*	%	*n*	%	*n*	%
Perceived level of knowledge on potential benefits and risks of psilocybin										
Good	6	30	22	46	20	56	4	24	52	43
Limited	14	70	26	54	16	44	13	76	69	57
Attitudes towards safety and efficacy of psilocybin-assisted therapy^a^										
Favourable	19	95	21	44	6	17	1	6	47	39
Non-favourable	1	5	27	56	30	83	16	94	74	61
Believes psilocybin-assisted therapy is a reasonable medical choice										
Yes	18	90	42	88	31	86	12	71	103	85
No	2	10	6	13	5	14	5	29	18	15
Perceived level of risk of psilocybin-assisted therapy on professional liability										
No/low risk	8	40	15	31	13	36	5	29	41	34
Moderate/high risk	7	35	17	35	14	39	9	53	47	39
Missing	5	25	16	33	9	25	3	18	33	27

a *p*-value <0.0001.

### Challenges to clinical integration

**Figure 1.**
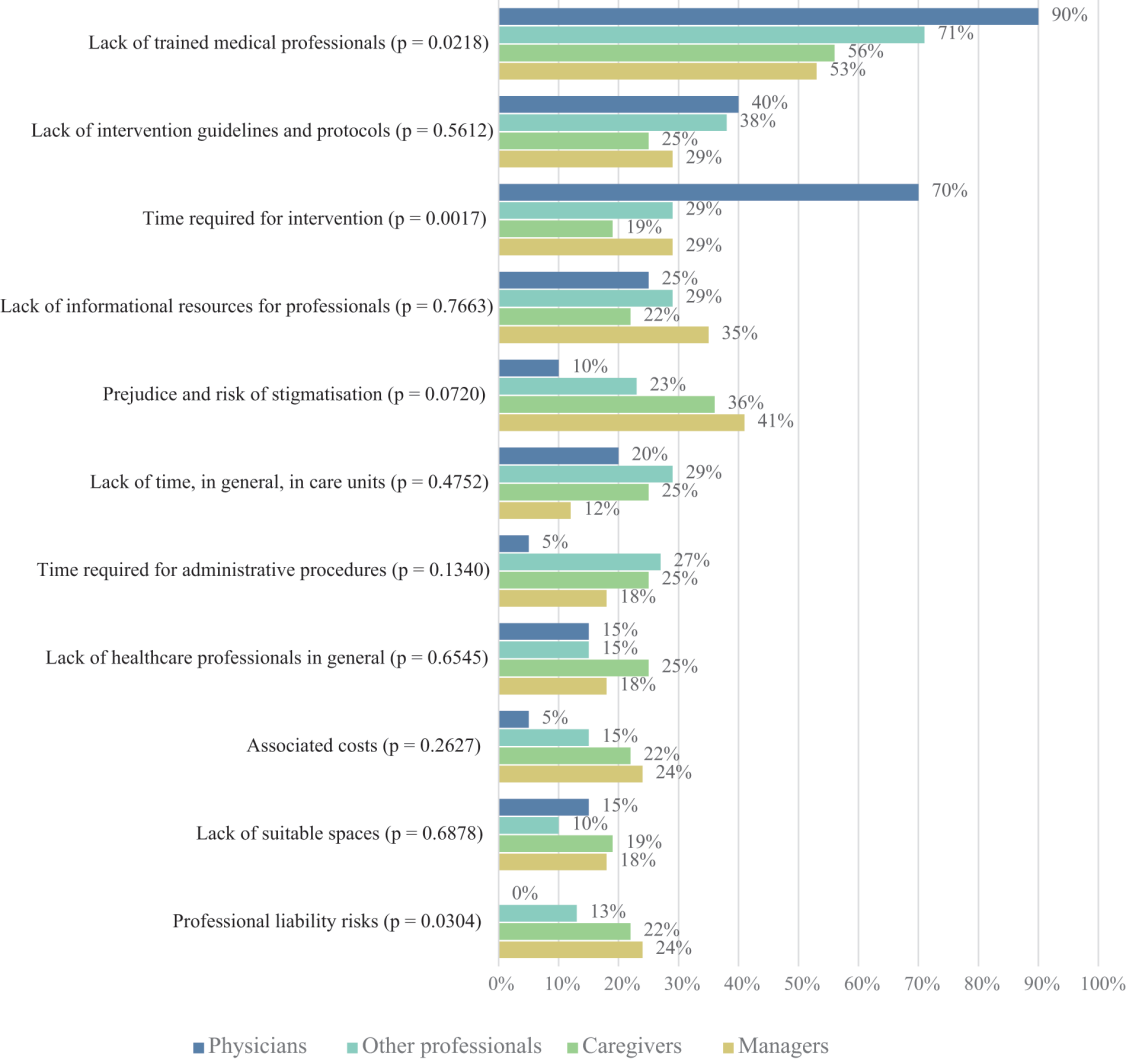
Perceived salience of key implementation barriers among stakeholders.

Figure 1 presents implementation barriers ranked by their perceived salience among stakeholders. Among these, the lack of trained medical professionals was the most widely recognised challenge to the clinical integration of psilocybin-assisted therapy. While broad consensus was observed across most items, statistically significant differences emerged in relation to three key challenges. Compared to other occupational groups, physicians placed greater emphasis on the lack of trained providers (*p* = 0.0218) and the time required for the intervention (*p* = 0.0017). In contrast, they expressed less concern about professional liability risks (*p* = 0.0304).

### Facilitators for an effective implementation

**Figure 2.**
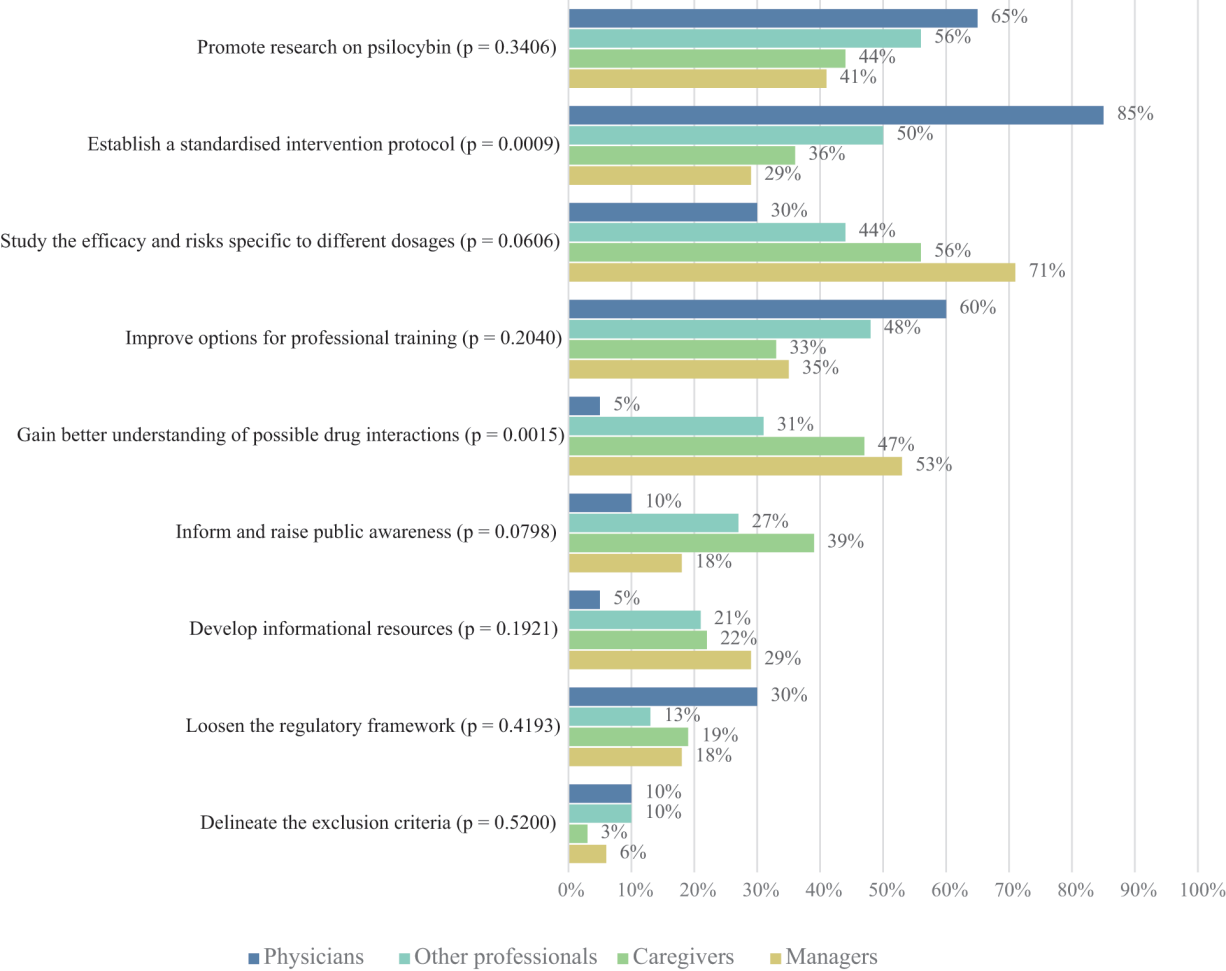
Perceived importance of key implementation facilitators among stakeholders.

Figure 2 shows actionable measures to facilitate the adoption and delivery of psilocybin-assisted therapy in palliative care settings, ranked by perceived importance. Stakeholders identified the promotion of research on psilocybin as the most important facilitator in their view. Physicians’ responses differed significantly from other stakeholders regarding the establishment of a standardised intervention protocol (*p* = 0.0009) and the need for better understanding of possible drug interactions (*p* = 0.0015). No significant difference between occupational groups was observed regarding the optimal timing for discussing psilocybin-assisted therapy with a patient.

**Figure 3.**
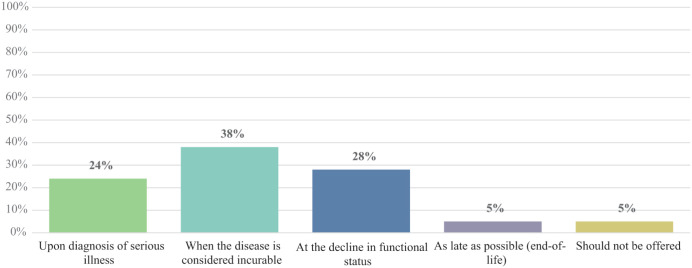
When should psilocybin-assisted therapy be discussed with a patient in the course of a serious and incurable illness?

As illustrated in Figure 3, 62% (*n* = 75) of stakeholders believed the intervention should be discussed early in the course of a life-threatening illness—either upon diagnosis (28%, *n* = 34) or once it is considered incurable (34%, *n* = 41).

**Figure 4.**
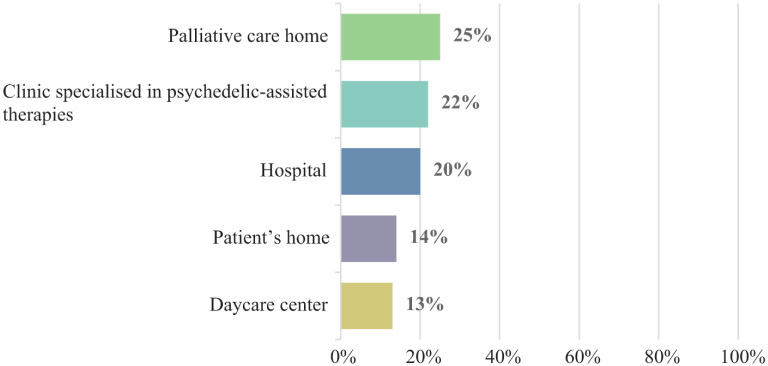
Which location do you think is most adequate for psilocybin-assisted therapy?

Furthermore, Figure 4 indicates stakeholders ranked palliative care homes and clinics specialising in psychedelic-assisted therapies, followed by hospitals, as the most appropriate settings for the provision of psilocybin-assisted therapy, with no significant difference between occupational groups.

## Discussion

### Main findings

This study assessed Canadian palliative care stakeholders’ knowledge and attitudes towards psilocybin-assisted therapy for existential distress, examined perceived barriers, and identified key facilitators for effective implementation. Physicians, other healthcare professionals, caregivers, and managers offered diverse perspectives on psilocybin-assisted therapy. Although less than half of participants reported having good knowledge about the potential benefits and risks of this intervention, most considered it as a reasonable approach for patients with life-threatening illnesses.

Attitudes towards the safety and clinical efficacy of psilocybin-assisted therapy in the context of palliative care varied significantly across occupational groups, with physicians expressing greater enthusiasm compared to caregivers and managers. Notably, stakeholders’ attitudes were not associated with perceived level of knowledge, but rather seemed to reflect role-specific concerns such as those related to patient safety, institutional risk, and public accountability. Although other healthcare professionals and caregivers reported higher perceived knowledge than physicians, they held less favourable views towards psilocybin-assisted therapy, indicating that familiarity with the topic is not a straightforward predictor of support. This finding illustrates how education alone, while necessary, is not sufficient to foster favourable attitudes. To be effective, educational and training initiatives should be complemented by dialogue addressing ethical, organisational, and medico-legal dimensions of psychedelic care.^47,48^

Furthermore, while participants largely concurred on the key challenges impeding implementation, physicians, who mostly worked in hospitals, placed greater emphasis on the shortage of trained medical practitioners (an issue previously identified as a primary concern among healthcare professionals^28^), as well as on the substantial time investment required for the intervention. Given the resource-intensive nature of the established model of psilocybin-assisted therapy,^49^ the practical barriers identified in our study warrant closer examination to support its sustainable integration within palliative care settings.

The need for further research into psilocybin’s efficacy and safety profile across varying dosages, the development of a standardised intervention protocol, and the scaling up of professional training opportunities emerged in our study as the most widely endorsed facilitators for psilocybin-assisted therapy’s integration into palliative medicine. These priorities align with conclusions drawn in previous studies,^22,23,33–36^ reinforcing their relevance to clinical implementation.

Our findings regarding the optimal timing and appropriate setting for delivery of psilocybin-assisted therapy call attention to crucial, context-specific considerations. A majority of stakeholders concurred that this therapeutic option should be introduced to patients during the earlier stages of the illness trajectory, either at the time of diagnosis of a serious condition or upon confirmation that the illness is incurable. This shared perspective closely aligns with a recent recommendation to consider the intervention “early in the course of a life-threatening illness when signiﬁcant anxio-depressive symptoms emerge, provided that the patient’s cognitive and psychological condition allows for informed consent and safe participation in treatment.”^23^ Furthermore, the establishment of dedicated facilities, supported by comprehensive logistics and organisational infrastructure, was proposed as a means to ensure the secure, well-regulated environment necessary for psilocybin-assisted therapy. Our findings reinforce this perspective, with stakeholders ranking specialised clinics as the second most suitable setting for delivering the intervention, following palliative care homes and ahead of hospitals.

Taken together, our findings underscore the need to foster interprofessional dialogue and develop tailored educational programmes that address the distinct informational needs and concerns of diverse stakeholder groups. Such efforts may not only harmonise perspectives on the safe and effective implementation of psilocybin-assisted therapy but also facilitate its seamless integration and promote equitable access within palliative care settings.

### What this study adds

This study engaged the nuanced perspectives of palliative care stakeholders, including the previously underrepresented caregivers and managers, and identified tangible implementation challenges alongside facilitators for psilocybin-assisted therapy. Of particular relevance, these findings are informed by the Canadian context, where its formal integration into healthcare settings is already underway.

Our results reveal a notable gap between perceived knowledge and overall acceptance of the intervention, suggesting that even in the absence of formal education or clinical training on the topic, a majority of stakeholders recognised psilocybin-assisted therapy as an acceptable treatment option. This level of acceptability is comparable to the findings of our previous, population-based survey of 2800 Canadians, in which 79% of respondents endorsed psilocybin-assisted therapy for patients approaching end-of-life.^46^ The higher acceptability rate observed in the present study may be attributed to the participants’ exposure to the realities of palliative care, as it was shown to significantly affect attitudes towards the intervention in our initial survey. Moreover, public and professional awareness of psilocybin-assisted therapy has increased manifestly over the 2-year interval between surveys.

Subgroup analyses indicate that clinical background and direct patient care responsibilities (as observed among physicians) are associated with more favourable attitudes, which may reflect a stronger sense of therapeutic agency, in contrast to the reticence expressed by caregivers and managers. That non-clinicians nonetheless regarded psilocybin-assisted therapy as a reasonable medical option is not contradictory, but rather points to values-based considerations, such as patient autonomy, informing their assessment of acceptability.

By addressing both practical barriers (e.g. trained medical provider scarcity, time constraints) and implementation strategies (e.g. protocol standardisation, early-stage consideration in incurable illness, development of specialised treatment centres), this study offers actionable insights for policymakers and healthcare institutions seeking to advance the clinical integration and equitable access to psilocybin-assisted therapy.

### Strengths and limitations

The use of a bilingual questionnaire and two complementary recruitment strategies across four Canadian provinces contributed to a diverse sample of individuals involved in palliative care. Stratification by occupational group further enhanced the interpretability of findings. A notable strength of this study is the inclusion of participants beyond traditional medical roles (such as beneficiary attendants, caregivers, volunteers, patient partners, and managers) whose perspectives on psilocybin-assisted therapy have been largely absent from existing discourse.

Despite these strengths, the study shares limitations common to web-based survey designs.^50^ Reliance on convenience sampling and participant self-selection may have introduced volunteer bias, particularly among individuals with prior interest or familiarity with psilocybin-assisted therapy. Additionally, the use of two distinct recruitment strategies may limit the comparability of occupational subgroups, and the sample size, while adequate for exploratory analysis, may not support generalisability. Future research should aim to recruit larger and more demographically diverse samples to enhance external validity and ensure more inclusive representation.

The decision to dichotomise certain survey items may have reduced sensitivity to mid-range responses and obscured nuanced variation in participant perspectives. While this approach facilitated clearer interpretation and statistical comparison, it may have oversimplified complex attitudes. Furthermore, although the questionnaire was intended to accommodate respondents regardless of their type of involvement in palliative care, certain items (such as the one pertaining to professional liability) may not have been uniformly understood. The relatively high proportion of missing data for such items across all occupational groups suggests that some concepts may have been perceived as difficult to assess or not relevant to some participants’ roles and should therefore be interpreted with caution.

Beyond these methodological considerations, perceived knowledge may not reliably reflect actual familiarity with the empirical evidence and may be subject to overestimation, as the quality and sources of information vary substantially across occupational groups. For instance, the certainty of evidence for psilocybin-assisted therapy as a treatment for existential distress in patients with life-threatening illnesses remains very low and may not be widely recognised, which could have influenced participants perceived knowledge and reported attitudes. Future studies would benefit from explicitly evaluating participants’ understanding of the certainty of evidence for such emerging interventions.

## Conclusion

Effective translation of the therapeutic potential of psilocybin-assisted therapy for existential distress from clinical trials into palliative medicine will require deliberate, structured stakeholder engagement. The heterogeneity of perspectives across occupational groups underscores the need for targeted educational interventions—an essential step towards fostering readiness, alignment, and safe integration into clinical practice.

As existential distress often emerges early in the illness trajectory, introducing psilocybin-assisted therapy at this stage may help relieve acute (often refractory) suffering and support lasting, meaningful improvements in overall quality of life. To ensure sustainable implementation, healthcare systems should prioritise standardised treatment protocols, expanded professional training, and dedicated delivery settings. Future research is needed to evaluate long-term outcomes, optimal dosing strategies, and the cost-effectiveness of early-stage models. Close collaboration among researchers, clinicians, policymakers, and patient communities will be critical to building the evidence base, defining best practices, and shaping policy frameworks that guarantee equitable access to psilocybin-assisted therapy for individuals with life-threatening illnesses.

## Supplemental Material

sj-docx-1-pmj-10.1177_02692163261446141 – Supplemental material for Implementing psilocybin-assisted therapy in palliative care settings: A survey of stakeholders Supplemental material, sj-docx-1-pmj-10.1177_02692163261446141 for Implementing psilocybin-assisted therapy in palliative care settings: A survey of stakeholders by Louis Plourde, Sue-Ling Chang, Olivia Nguyen, Nicolas Garel, Houman Farzin, Jean-François Stephan, Jean-Sébastien Fallu and Michel Dorval in Palliative Medicine

sj-docx-2-pmj-10.1177_02692163261446141 – Supplemental material for Implementing psilocybin-assisted therapy in palliative care settings: A survey of stakeholders Supplemental material, sj-docx-2-pmj-10.1177_02692163261446141 for Implementing psilocybin-assisted therapy in palliative care settings: A survey of stakeholders by Louis Plourde, Sue-Ling Chang, Olivia Nguyen, Nicolas Garel, Houman Farzin, Jean-François Stephan, Jean-Sébastien Fallu and Michel Dorval in Palliative Medicine

sj-docx-3-pmj-10.1177_02692163261446141 – Supplemental material for Implementing psilocybin-assisted therapy in palliative care settings: A survey of stakeholders Supplemental material, sj-docx-3-pmj-10.1177_02692163261446141 for Implementing psilocybin-assisted therapy in palliative care settings: A survey of stakeholders by Louis Plourde, Sue-Ling Chang, Olivia Nguyen, Nicolas Garel, Houman Farzin, Jean-François Stephan, Jean-Sébastien Fallu and Michel Dorval in Palliative Medicine
