# Supplementary material for: Implementing psilocybin-assisted therapy in palliative care settings: A survey of stakeholders
Source: Palliat Med. 2026 May 19;40(7):1047–58. doi: 10.1177/02692163261446141 (PMC13323913; doi:10.1177/02692163261446141)
Supplement: sj-docx-1-pmj-10.1177_02692163261446141 – Supplemental material for Implementing psilocybin-assisted therapy in palliative care settings: A survey of stakeholders [file sj-docx-1-pmj-10.1177_02692163261446141.docx]

| **Table 1S.** Descriptive statistics for Sample 1 and Sample 2 | | | | | | |
| --- | --- | --- | --- | --- | --- | --- |
|  | **Sample 1** | | **Sample 2** | | **Global** | |
|  | n | (%) | n | (%) | n | (%) |
| Total | 43 | (100) | 78 | (100) | 121 | (100) |
| ***Occupation ***** | | | | | | |
| Physician | 20 | (47) | 0 | (0) | 20 | (17) |
| Other professional | 20 | (47) | 28 | (36) | 48 | (40) |
| Caregiver | 3 | (7) | 33 | (42) | 36 | (30) |
| Manager | 0 | (0) | 17 | (22) | 17 | (14) |
| ***Province ***** | | | | | | |
| Québec | 43 | (100) | 22 | (28) | 65 | (54) |
| Ontario | 0 | (0) | 22 | (28) | 22 | (18) |
| Alberta | 0 | (0) | 14 | (18) | 14 | (12) |
| British Columbia | 0 | (0) | 20 | (26) | 20 | (17) |
| ***Age*** | | | | | | |
| 18–34 | 9 | (21) | 17 | (22) | 26 | (21) |
| 35–54 | 20 | (47) | 33 | (42) | 53 | (44) |
| ≥ 55 | 14 | (33) | 28 | (36) | 42 | (35) |
| ***Gender **** | | | | | | |
| Woman | 32 | (74) | 48 | (62) | 80 | (66) |
| Man | 10 | (23) | 29 | (37) | 39 | (32) |
| Non-binary | 0 | (0) | 1 | (1) | 1 | (1) |
| Missing | 1 | (2) | - | - | 1 | (1) |
| ***Years involved in the field of palliative care **** | | | | |  |  |
| 0–2 | 6 | (14) | 27 | (35) | 33 | (27) |
| 3–10 | 15 | (35) | 27 | (35) | 42 | (35) |
| > 10 | 22 | (51) | 18 | (23) | 40 | (33) |
| Missing | - | - | 6 | (8) | 6 | (5) |
| ***Primarily involved in **** | | | | | |  |
| Hospital | 19 | (44) | 23 | (29) | 42 | (35) |
| Outpatient clinic | 6 | (14) | 1 | (1) | 7 | (6) |
| Palliative care home | 7 | (16) | 6 | (8) | 13 | (11) |
| Patients' homes | 9 | (21) | 21 | (27) | 30 | (25) |
| Long-term care center | 0 | (0) | 6 | (8) | 6 | (5) |
| Academia | 0 | (0) | 4 | (5) | 4 | (3) |
| Missing | 2 | (5) | 17 | (22) | 19 | (16) |
| ***Approximate number of hours per week in contact with people in palliative care ***** | | | | | | |
| 0 | 1 | (2) | 18 | (23) | 19 | (16) |
| 1–10 | 6 | (14) | 31 | (40) | 37 | (31) |
| 11–20 | 12 | (28) | 12 | (15) | 24 | (20) |
| > 20 | 23 | (53) | 12 | (15) | 35 | (29) |
| Missing | 1 | (2) | 5 | (6) | 6 | (5) |
| ***Lifetime psilocybin use*** | | | | | | |
| Yes | 10 | (23) | 19 | (24) | 29 | (24) |
| No | 32 | (74) | 58 | (74) | 90 | (74) |
| Missing | 1 | (2) | 1 | (1) | 2 | (2) |
| ** = p-value ≤ 0.05*  *** = p-value < 0.0001* | | | | | | |
