# Supplementary material for: Implementing psilocybin-assisted therapy in palliative care settings: A survey of stakeholders
Source: Palliat Med. 2026 May 19;40(7):1047–58. doi: 10.1177/02692163261446141 (PMC13323913; doi:10.1177/02692163261446141)
Supplement: sj-docx-2-pmj-10.1177_02692163261446141 – Supplemental material for Implementing psilocybin-assisted therapy in palliative care settings: A survey of stakeholders [file sj-docx-2-pmj-10.1177_02692163261446141.docx]

| **Table 2S.** Detailed Likert score distributions and composite measures for perceived knowledge and attitudes | | | | | | | | | | |
| --- | --- | --- | --- | --- | --- | --- | --- | --- | --- | --- |
|  | **Physicians** | | **Other professionals** | | **Caregivers** | | **Managers** | | **Global** | |
|  |  |  |  |  |  |  |  |  |  |  |
|  | n | (%) | n | (%) | n | (%) | n | (%) | n | (%) |
| Total | 20 | (100) | 48 | (100) | 36 | (100) | 17 | (100) | 121 | (100) |
| ***1- I consider myself to have a good knowledge of the potential benefits of psilocybin for medical purposes.*** *[p = 0.1609]* | | | | | | | | | | |
| 1 - Strongly disagree | 2 | (10) | 9 | (19) | 2 | (6) | 1 | (6) | 14 | (12) |
| 2 - Disagree | 6 | (30) | 6 | (13) | 5 | (14) | 9 | (53) | 26 | (21) |
| 3 - Neither agree, nor disagree | 4 | (20) | 12 | (25) | 10 | (28) | 3 | (18) | 29 | (24) |
| 4 - Agree | 4 | (20) | 17 | (35) | 12 | (33) | 2 | (12) | 35 | (29) |
| 5 - Strongly agree | 4 | (20) | 4 | (8) | 7 | (19) | 2 | (12) | 17 | (14) |
| *Mean (SD)* | *3.10 (1.33)* | | *3.02 (1.26)* | | *3.47 (1.13)* | | *2.71 (1.16)* | | *3.12 (1.24)* | |
| ***2- I consider myself to have a good knowledge of the potential risks of psilocybin.*** *[p = 0.0663]* | | | | | | | | | | |
| 1 - Strongly disagree | 2 | (10) | 6 | (13) | 1 | (3) | 1 | (6) | 10 | (8) |
| 2 - Disagree | 9 | (45) | 15 | (31) | 4 | (11) | 6 | (35) | 34 | (28) |
| 3 - Neither agree, nor disagree | 4 | (20) | 7 | (15) | 15 | (42) | 7 | (41) | 33 | (27) |
| 4 - Agree | 2 | (10) | 18 | (38) | 11 | (31) | 2 | (12) | 33 | (27) |
| 5 - Strongly agree | 3 | (15) | 2 | (4) | 5 | (14) | 1 | (6) | 11 | (9) |
| *Mean (SD)* | *2.75 (1.25)* | | *2.90 (1.17)* | | *3.42 (0.97)* | | *2.76 (0.97)* | | *3.01 (1.12)* | |
| ***[1,2] Perceived knowledge composite score*** *[p = 0.0940]* | | | | | | | | | | |
| *Mean (SD)* | *5.85 (2.48)* | | *5.92 (2.32)* | | *6.89 (2.00)* | | *5.47 (1.94)* | | *6.13 (2.24)* | |
| ***3- Psilocybin can be safe under medical supervision.*** *[p < 0.0001]* | | | | | | | | | | |
| 1 - Strongly disagree | 0 | (0) | 3 | (6) | 4 | (11) | 2 | (12) | 9 | (7) |
| 2 - Disagree | 0 | (0) | 15 | (31) | 14 | (39) | 8 | (47) | 37 | (31) |
| 3 - Neither agree, nor disagree | 3 | (15) | 13 | (27) | 14 | (39) | 5 | (29) | 35 | (29) |
| 4 - Agree | 10 | (50) | 13 | (27) | 0 | (0) | 1 | (6) | 24 | (20) |
| 5 - Strongly agree | 7 | (35) | 4 | (8) | 4 | (11) | 1 | (6) | 16 | (13) |
| *Mean (SD)* | *4.20 (0.70)* | | *3.00 (1.09)* | | *2.61 (1.08)* | | *2.47 (1.01)* | | *3.01 (1.16)* | |
| ***4- It is important to investigate how psilocybin-assisted therapy can be integrated into healthcare settings.*** *[p < 0.0001]* | | | | | | | | | | |
| 1 - Strongly disagree | 0 | (0) | 4 | (8) | 8 | (22) | 3 | (18) | 15 | (12) |
| 2 - Disagree | 1 | (5) | 17 | (35) | 15 | (42) | 9 | (53) | 42 | (35) |
| 3 - Neither agree, nor disagree | 0 | (0) | 8 | (17) | 8 | (22) | 5 | (29) | 21 | (17) |
| 4 - Agree | 5 | (25) | 10 | (21) | 0 | (0) | 0 | (0) | 15 | (12) |
| 5 - Strongly agree | 14 | (70) | 9 | (19) | 5 | (14) | 0 | (0) | 28 | (23) |
| *Mean (SD)* | *4.60 (0.75)* | | *3.06 (1.29)* | | *2.42 (1.25)* | | *2.12 (0.70)* | | *2.99 (1.38)* | |
| ***5- Psilocybin-assisted therapy can alleviate existential distress.*** *[p < 0.0001]* | | | | | | | | | | |
| 1 - Strongly disagree | 0 | (0) | 3 | (6) | 5 | (14) | 3 | (18) | 11 | (9) |
| 2 - Disagree | 0 | (0) | 12 | (25) | 12 | (33) | 6 | (35) | 30 | (25) |
| 3 - Neither agree, nor disagree | 4 | (20) | 18 | (38) | 15 | (42) | 7 | (41) | 44 | (36) |
| 4 - Agree | 8 | (40) | 11 | (23) | 1 | (3) | 1 | (6) | 21 | (17) |
| 5 - Strongly agree | 8 | (40) | 4 | (8) | 3 | (8) | 0 | (0) | 15 | (12) |
| *Mean (SD)* | *4.20 (0.77)* | | *3.02 (1.04)* | | *2.58 (1.05)* | | *2.35 (0.86)* | | *2.99 (1.14)* | |
| ***6- Psilocybin-assisted therapy may be more effective than conventional approaches in treating existential distress.*** *[p < 0.0001]* | | | | | | | | | | |
| 1 - Strongly disagree | 0 | (0) | 3 | (6) | 4 | (11) | 7 | (41) | 14 | (12) |
| 2 - Disagree | 0 | (0) | 12 | (25) | 11 | (31) | 3 | (18) | 26 | (21) |
| 3 - Neither agree, nor disagree | 10 | (50) | 25 | (52) | 16 | (44) | 6 | (35) | 57 | (47) |
| 4 - Agree | 4 | (20) | 4 | (8) | 2 | (6) | 1 | (6) | 11 | (9) |
| 5 - Strongly agree | 6 | (30) | 4 | (8) | 3 | (8) | 0 | (0) | 13 | (11) |
| *Mean (SD)* | *3.80 (0.89)* | | *2.88 (0.96)* | | *2.69 (1.04)* | | *2.06 (1.03)* | | *2.86 (1.09)* | |
| ***[3-6] Attitudes composite score*** *[p < 0.0001]* | | | | | | | | | | |
| *Mean (SD)* | *16.80 (2.38)* | | *11.96 (4.02)* | | *10.31 (4.07)* | | *9.00 (2.76)* | | *11.85 (4.36)* | |
