# Supplementary material for: Implementing psilocybin-assisted therapy in palliative care settings: A survey of stakeholders
Source: Palliat Med. 2026 May 19;40(7):1047–58. doi: 10.1177/02692163261446141 (PMC13323913; doi:10.1177/02692163261446141)
Supplement: sj-docx-3-pmj-10.1177_02692163261446141 – Supplemental material for Implementing psilocybin-assisted therapy in palliative care settings: A survey of stakeholders [file sj-docx-3-pmj-10.1177_02692163261446141.docx]

**Survey of people involved in palliative care on the use of psilocybin-assisted therapy to treat existential distress**

**[Background]** In this questionnaire, we use the definition of **palliative care** proposed by the *International Association for Hospice and Palliative Care*, which refers to “active holistic care of individuals across all ages with serious health-related suffering due to severe illness, and especially of those near the end of life. It aims to improve the quality of life of patients, their families and their caregivers.” Palliative care includes “prevention, early identification, comprehensive assessment and management of physical issues, including pain and other distressing symptoms, psychological distress, spiritual distress and social needs.”

**1. What is your current occupation?**

1. Physician
2. Nurse clinician
3. Licensed practical nurse
4. Specialised nurse practitioner
5. Psychiatrist
6. Psychologist
7. Psychotherapist
8. Pharmacist
9. Physiotherapist
10. Occupational therapist
11. Spiritual care provider
12. Social worker
13. Beneficiary attendant
14. Caregiver
15. Volunteer
16. Manager / Administrator
17. Researcher
18. Student
19. Patient partner
20. Other (please specify) [open box]

**2. How many years have you been involved in the field of palliative care?**

1. 0-2 years
2. 3-5 years
3. 6-10 years
4. 11-20 years
5. More than 20 years

**3. What type of environment** **are you primarily involved in?**

1. Hospital
2. Outpatient clinic
3. Palliative care home
4. Patients' homes
5. Long-term care facility
6. Academia
7. Other (please specify) [open box]
8. Not applicable

**4. Approximately how many hours per week are you in contact with people in palliative care?**

1. 0
2. 1-10
3. 11-20
4. 21-30
5. More than 30
6. Don't know / Prefer not to answer

**[Background]** In this questionnaire, **existential distress** refers to a disorder affecting people facing a serious and incurable illness as well as the prospect of their own death. Still poorly understood and particularly difficult to treat, this complex condition can encompass symptoms of depression, anxiety, demoralisation, and loss of meaning. Conventional intervention approaches to treat existential distress include pharmacotherapy, psychotherapy, and spiritual accompaniment.

**5. What is your level of exposure to the existential distress of people facing a serious and incurable illness?**

1. Very low exposure
2. Low exposure
3. Moderately exposed
4. Highly exposed
5. Don't know / Prefer not to answer

**6. In your opinion, to what extent is existential distress a common condition among people in palliative care?**

1. Very common
2. Somewhat common
3. Uncommon
4. Very uncommon
5. Don't know / Prefer not to answer

**7. In your opinion, to what extent is existential distress adequately identified in the current context of palliative care?**

1. Very adequately
2. Somewhat adequately
3. Inadequately
4. Very inadequately
5. Don't know / Prefer not to answer

**8. In your opinion, to what extent is existential distress effectively managed with current intervention approaches?**

1. Very effectively
2. Somewhat effectively
3. Ineffectively
4. Very ineffectively
5. Don't know / Prefer not to answer

**9. In your opinion, can the suffering caused by existential distress motivate people to hasten their death by requesting medical assistance in dying?**

1. Yes
2. No
3. Don't know / Prefer not to answer

**[Background]** Since its amendment in January 2022, Health **Canada's Special Access Program** allows physicians to apply for access to psilocybin—the psychoactive constituent of "magic mushrooms"—to treat the existential distress of people with a serious and incurable illness. The use of this substance, which remains otherwise prohibited, can only be authorised when conventional therapies to treat existential distress have failed or are not suitable for the patient's profile. These requests are assessed on a case-by-case basis.

**10. Have you heard of Health Canada's Special Access Program?**

1. Yes
2. No
3. Don't know / Prefer not to answer

**11. If so, how did you hear about it? (check all that apply)**

1. In the media (newspapers, magazines, television, radio)
2. In scientific publications
3. On the Internet (websites, social networks, discussion forums)
4. Through friends, family members or acquaintances
5. Through a healthcare professional
6. Through a patient or a relative
7. Other (please specify) [open box]
8. Don't know / Prefer not to answer

**12. Based on your current knowledge, what are the levels of risk associated with taking psilocybin?** [Table with scale: No risk; Low risk; Moderate risk; High risk; Don't know]

- Headaches
- Dependence or addiction?
- Brain damage
- Damage to genetic material
- Heart problems
- Overdose
- Panic episode / difficult experience
- Onset or aggravation of mental disorders
- Risky behaviours
- Cognitive impairment (memory, language, reasoning)
- Persistent psychotic disorder after treatment

**13. Have you ever used psilocybin? (check all that apply)**

1. Yes, for therapeutic purposes
2. Yes, for recreational purposes
3. Yes, in microdoses
4. Yes, other (please specify) [open box]
5. No
6. Prefer not to answer

**[Background] Psilocybin-assisted therapy** is based on the administration of a high dose of psilocybin in a controlled environment, under the guidance of two specially trained healthcare professionals. It aims to bring about a profound and meaningful psychic experience, where important realisations can be transformative. Lasting six to eight hours, the psychedelic session is preceded by preparation meetings and followed by integration sessions, which play a determining role in the psychotherapeutic process. This approach is likely to result in a rapid, substantial, and lasting reduction in symptoms of existential distress. In some cases, this process can be repeated for optimal symptom relief.

**14. Do you believe psilocybin-assisted therapy is a reasonable medical choice for someone with a serious and incurable illness suffering from existential distress?**

1. Yes, since it is a personal choice
2. Yes, but only if other treatments for existential distress have proven ineffective
3. Yes, for another reason (please specify) [open box]
4. No (please specify) [open box]
5. Uncertain

**15. Have you ever been approached by a patient or someone close about psilocybin-assisted therapy?**

1. Yes
2. No
3. Not applicable / Prefer not to respond

**16. If a patient confided in you that they were considering** **psilocybin-assisted therapy, what would be your reaction?**

1. Very favourable
2. Favourable
3. Neither favourable nor unfavourable
4. Unfavourable
5. Very unfavourable
6. Not applicable

**17. In your opinion, if you approached a patient about the possibility of using psilocybin, what would be their reaction, in general?**

1. Very favourable
2. Favourable
3. Neither favourable nor unfavourable
4. Unfavourable
5. Very unfavourable
6. Not applicable

**18. If you were to tell your professional or academic circle that you were considering integrating psilocybin-assisted therapy into your practice, what would be the general reaction?**

1. Very favourable
2. Favourable
3. Neither favourable nor unfavourable
4. Unfavourable
5. Very unfavourable
6. Not applicable

**19. What is your level of agreement on the following statements?** [Scale: Strongly disagree; Disagree; Neither agree, nor disagree; Agree; Strongly agree]

- I consider myself to have a good knowledge of the potential benefits of psilocybin for medical purposes.
- I consider myself to have a good knowledge of the potential risks of psilocybin.
- Psilocybin can be safe under medical supervision.
- It is important to investigate how psilocybin-assisted therapy can be integrated into healthcare settings.
- Psilocybin-assisted therapy can alleviate existential distress.
- Psilocybin-assisted therapy may prove to be more effective than conventional approaches (pharmacotherapy, psychotherapy, spiritual accompaniment) in treating existential distress.

**20. When should psilocybin-assisted therapy be discussed with a person in the course of a serious and incurable illness?**

1. Upon diagnosis of serious illness
2. When the disease is considered incurable
3. At the decline in functional status
4. As late as possible (terminally ill / end-of-life)
5. Should not be offered

**21. What do you think are the risk levels of psilocybin-assisted therapy that are specific to people with an incurable disease?** [Table with scale: No risk; Low risk; Moderate risk; High risk; Don't know]

- Contraindication(s)/interactions with concurrent treatments
- Panic episode(s)
- Worsening of psychic/psychological condition
- Worsening of physical condition
- Dependence
- No clinical effect
- Other (please specify) [open box]

**22. Which of the following measures do you consider most important to facilitate the integration of psilocybin-assisted therapy into the context of palliative care?** (check 3 choices)

1. Establish a standardised intervention protocol
2. Promote research on psilocybin
3. Loosen the regulatory framework
4. Study the efficacy and risks specific to different dosages
5. Develop informational resources
6. Delineate the exclusion criteria
7. Improve options for professional training
8. Inform and raise awareness among the population
9. Gain a better understanding of possible drug interactions
10. Other (please specify) [open box]

**23. What do you see as the key issues related to the introduction of** **psilocybin-assisted therapy in the context of palliative care?** (check 3 choices)

1. Lack of trained medical professionals for psilocybin-assisted therapy
2. Lack of healthcare professionals in general
3. Lack of informational resources for healthcare professionals
4. Time required for administrative procedures with Health Canada
5. Time required for psilocybin-assisted therapy (preparation, assisted session, integration)
6. Lack of intervention guidelines and protocols
7. Lack of suitable spaces for psychedelic sessions
8. Lack of time, in general, in care units
9. Prejudice and risk of stigmatisation
10. Professional liability risks
11. Costs associated with therapy
12. Other (please specify) [open box]

**24. According to you, what is the level of risk that may result from the practice of psilocybin-assisted therapy in terms of professional liability?** [Scale: No Risk; Low risk; Moderate risk; High risk; Don't know]

**[Background]** In the current context, only healthcare professionals who can prescribe medications (physicians and specialised nurse practitioners) can apply for access to psilocybin through the Special Access Program. If necessary, a licensed physician may then mandate two co-therapists to take over the psilocybin-assisted therapy process with the patient involved in the request.

**25. In your opinion, which of those involved in palliative care might be well-suited (after completing accredited training) to act as co-therapists in psilocybin-assisted therapy?** (check all that apply)

1. Physician
2. Psychiatrist
3. Psychologist
4. Psychotherapist
5. Pharmacist
6. Nurse
7. Social worker
8. Spiritual care provider
9. Physiotherapist
10. Occupational therapist
11. Volunteer
12. Caregiver
13. Patient partner
14. Other (please specify) [open box]

**26. Which location do you think is most adequate for psilocybin-assisted therapy?**

1. Hospital
2. Palliative care home
3. Daycare centre
4. Community organisation supporting people with cancer
5. Patient’s home
6. Clinic specialised in psychedelic-assisted therapies
7. Other (please specify) [open box]

**27. Do you think it would be beneficial to consider the use of psilocybin for medical purposes as part of group therapy, rather than as a stand-alone therapy?**

1. Yes, absolutely
2. Yes, possibly
3. No, not really
4. No, absolutely not
5. Uncertain

**28. Do you think that psilocybin-assisted therapy could also be offered to treat existential distress among caregivers?**

1. Yes
2. No
3. Uncertain

**29. What is your level of agreement on the following statements?** [Scale: Strongly disagree; Disagree; Neither agree, nor disagree; Agree; Strongly agree + Not applicable]

- I would be interested in taking a *general* training on psilocybin-assisted therapy.
- I would be interested in taking an *accredited* training in psilocybin-assisted therapy.
- I would be interested in incorporating psilocybin-assisted therapy into my practice.
- Healthcare professionals should have experienced psilocybin as part of their training in order to offer assisted therapy.
- Psilocybin should be legalised for medical purposes.

**30. What topics would you like to learn more about?** (check two choices)

1. Pharmacology of psilocybin
2. Clinical efficacy of psilocybin
3. Risks associated with psilocybin
4. Managing difficult experiences
5. Exclusion criteria for patients
6. Possible psilocybin drug interactions
7. How to offer psilocybin-assisted therapy
8. Other (please specify) [open box]

**31. What sources of information about psilocybin-assisted therapy would you trust the most?** (check two choices)

1. Professional orders
2. Professional associations
3. Academic research centres
4. Pharmaceutical companies
5. Experienced healthcare professionals
6. Private organisations providing accredited training
7. Scientific literature
8. Other(s) (please specify) [open box]

**32. How old are you?**

1. 18-24 years old
2. 25-34 years old
3. 35-44 years
4. 45-54 years old
5. 55 and older

**33. What is your gender?** (Gender refers to the current gender, which may differ from the sex assigned at birth or the sex recorded in legal documents.)

1. Woman
2. Man
3. Non-binary
4. Other (please specify) [open box]
5. Prefer not to answer

**34. Where do you place your political orientation on the following scale?** [5-point Likert scale: Conservative; Somewhat conservative; Neutral; Somewhat progressive; Progressive + Don't know / Prefer not to answer]

**35. Are there any topics that have not been addressed in this questionnaire that you would like to see further considered and discussed?** [open box]
